# A missense *GDF5* variant causes brachydactyly type A1 and multiple‐synostoses syndrome 2

**DOI:** 10.1002/jsp2.1302

**Published:** 2023-11-28

**Authors:** Juyi Li, Xiaofang Liang, Xiufang Wang, Pei Yang, Xiaofei Jian, Lei Fu, Aiping Deng, Chao Liu, Jianxin Liu

**Affiliations:** ^1^ Department of Pharmacy, The Central Hospital of Wuhan Tongji Medical College, Huazhong University of Science and Technology Wuhan Hubei China; ^2^ Department of Dermatology, The Central Hospital of Wuhan Tongji Medical College, Huazhong University of Science and Technology Wuhan China; ^3^ Department of Pain, The Central Hospital of Wuhan Tongji Medical College, Huazhong University of Science and Technology Wuhan Hubei China; ^4^ Department of Radiology, The Central Hospital of Wuhan Tongji Medical College, Huazhong University of Science and Technology Wuhan Hubei China; ^5^ Department of Orthopedics, The Central Hospital of Wuhan Tongji Medical College, Huazhong University of Science and Technology Wuhan Hubei China; ^6^ Department of Ultrasound, The Central Hospital of Wuhan Tongji Medical College, Huazhong University of Science and Technology Wuhan Hubei China; ^7^ Hubei Key Laboratory of Diabetes and Angiopathy Hubei University of Science and Technology Xianning Hubei China

**Keywords:** brachydactyly type A1, GDF5, multiple‐synostoses syndrome 2, variant, whole‐exome sequencing

## Abstract

**Objective:**

This study aimed to identify the molecular defects and clinical manifestations in a Chinese family with brachydactyly (BD) type A1 (BDA1) and multiple‐synostoses syndrome 2 (SYNS2).

**Methods:**

A Chinese family with BDA1 and SYNS2 was enrolled in this study. Whole‐exome sequencing was used to analyze the gene variants in the proband. The sequences of the candidate pathogenic variant in *GDF5* was validated via Sanger sequencing. I‐TASSER and PyMOL were used to analyze the functional domains of the corresponding mutant proteins.

**Results:**

The family was found to have an autosomal‐dominantly inherited combination of BDA1 and SYNS2 caused by the S475N variant in the *GDF5* gene. The variant was located within the functional region, and the mutated residue was found to be highly conserved among species. Via bioinformatic analyses, we predicted this variant to be deleterious, which perturb the protein function. The substitution of the negatively charged amino acid S475 with the neutral N475 was predicted to disrupt the formation of salt bridges with Y487 and impair the structure, stability, and function of the protein, consequently, the abnormalities in cartilage and bone development ensue.

**Conclusions:**

A single genetic variant (S475N) which disrupt the formation of salt bridges with Y487, in the interface of the antagonist‐ and receptor‐binding sites of GDF5 concurrently causes two pathological mechanisms. This is the first report of this variant, identified in a Chinese family with BDA1 and SYNS2.

## Graphical abstract

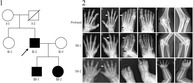

## 1 INTRODUCTION

Growth differentiation factor 5 (GDF5) is a member of the family of bone morphogenetic proteins (BMPs), which belongs to the transforming growth factor beta (TGFβ) superfamily.^1^ GDF5 plays an important role in limb development. It determines the sizes of the initial cartilaginous condensations during joint development.^2^ GDF5 regulates early chondrogenesis by interacting with two different types of BMP 1 receptors, namely BMPR1A and BMPR1B, with a higher affinity for BMPR1B among the two.^3^ BMP antagonists, such as NOGGIN (NOG), inhibit GDF5 activity and GDF5‐induced signal transduction by blocking the receptor‐binding site of GDF5.^4^ A variant of *GDF5* is associated with various genetic diseases, such as osteogenesis imperfecta and chondrodysplasia.^5^, ^6^, ^7^, ^8^, ^9^, ^10^, ^11^, ^12^ Some loss‐of‐function *GDF5* variants result in reduced osteogenesis or abnormal bone development, such as brachydactyly (BD), whereas some gain‐of‐function *GDF5* variants cause proximal symphalangism (SYM1, OMIM #185800) and multiple‐synostoses syndrome 2 (SYNS2, OMIM #610017). BD is classified into five types (A–E) according to the affected phalanges, and type A comprises three sub‐types (A1–A3).^13^ A variant of GDF5 has been shown to be related to BDA1 (OMIM #112500), BDA2 (OMIM #112600), and BDC (OMIM #113100).^6^, ^14^, ^15^

Homozygous deletion variants in *GDF5* lead to the development of extremely short fingers and limbs, which is associated with various types of acrochondrodysplasia.^16^, ^17^ In contrast to loss‐of‐function *GDF5* variants, gain‐of‐function *GDF5* variants increase the chondrogenous activity and are associated with SYM1 and SYNS2.^6^, ^17^ SYM1 is characterized by proximal interphalangeal stiffness, in which the carpus and tarsus are fused. A variant of *GDF5* causes joint adhesion in the elbows and knees, which is a feature of SYNS2.

Here, we describe a Chinese family with concurrent clinical features of BDA1 and SYNS2, which we found to result from a variant in the mature domain of GDF5 (S475N).

## 2 MATERIALS AND METHODS

All the participants completed questionnaires regarding their medical and family history, and the collected information was supplemented by medical records. The diagnosis and classification of BDA1 and SYNS2 complied with the latest guidelines.^16^ All the participants signed informed consent for this study, which was approved by the Ethics Committee of the Central Hospital of Wuhan.

### 2.1 DNA extraction and whole‐exome sequencing (WES)

The clinical data of the family members were obtained, and 5 mL of fasting blood of each member was collected into a tube containing EDTA as the anticoagulant. Genomic DNA was isolated from these peripheral‐blood samples by using a DNA Extraction Kit (TIANGEN, Beijing, China) according to the instructions of the manufacturer.^18^ WES was performed on the proband. Agilent SureSelect Human All Exon V6 kit was used to capture the genomic sequences, followed by sequencing on the Illumina hiSeq2500 System. The reference genome was human GRCh37/hg19.

### 2.2 Molecular genetic analyses

The single‐nucleotide polymorphism (SNP) and insertion–deletion (InDel) variants in the proband were identified by comparing the obtained genomic sequences with those in the following publicly available databases: 1000 Genomes, Exome Aggregation Consortium, Single‐Nucleotide Polymorphism Database, NHLBI Exome Sequencing Project, and Genome Aggregation Database. Several in silico prediction tools (SIFT, PolyPhen2_HVAR, Polyphen2_HDIV, LRT, and FATHMM) were used to predict the effects of identified variants on protein structure and function.

### 2.3 Sanger sequencing

Sanger sequencing was performed to confirm that the proband and his family carried the candidate variant in *GDF5*. The target DNA segments were amplified via polymerase chain reaction (PCR). The primers for *GDF5* were as follows: forward primer, 5′‐ GCAGACGGGCAGCAATCC‐3′; and reverse primer, 5′‐ CAAGCGACCCAGCAAGAACC‐3′. The PCR conditions were as follows: 95°C for 5 min; 35 cycles of 95°C for 1 min, 59°C for 30 s, then 72°C for 10 min. After the PCR product was purified, it was sequenced using an ABI3730XL Automatic Sequencer (Applied Biosystems, Foster City, CA).

### 2.4 Structural modeling

I‐TASSER (https://seq2fun.dcmb.med.umich.edu//I-TASSER/) and PyMOL (https://pymol.org/2/) were used to simulate the three‐dimensional (3D) structure of the GDF5 protein and analyze the structural difference between the wild‐type and mutant proteins.

## 3 RESULTS

### 3.1 Clinical characteristics

**FIGURE 1:**
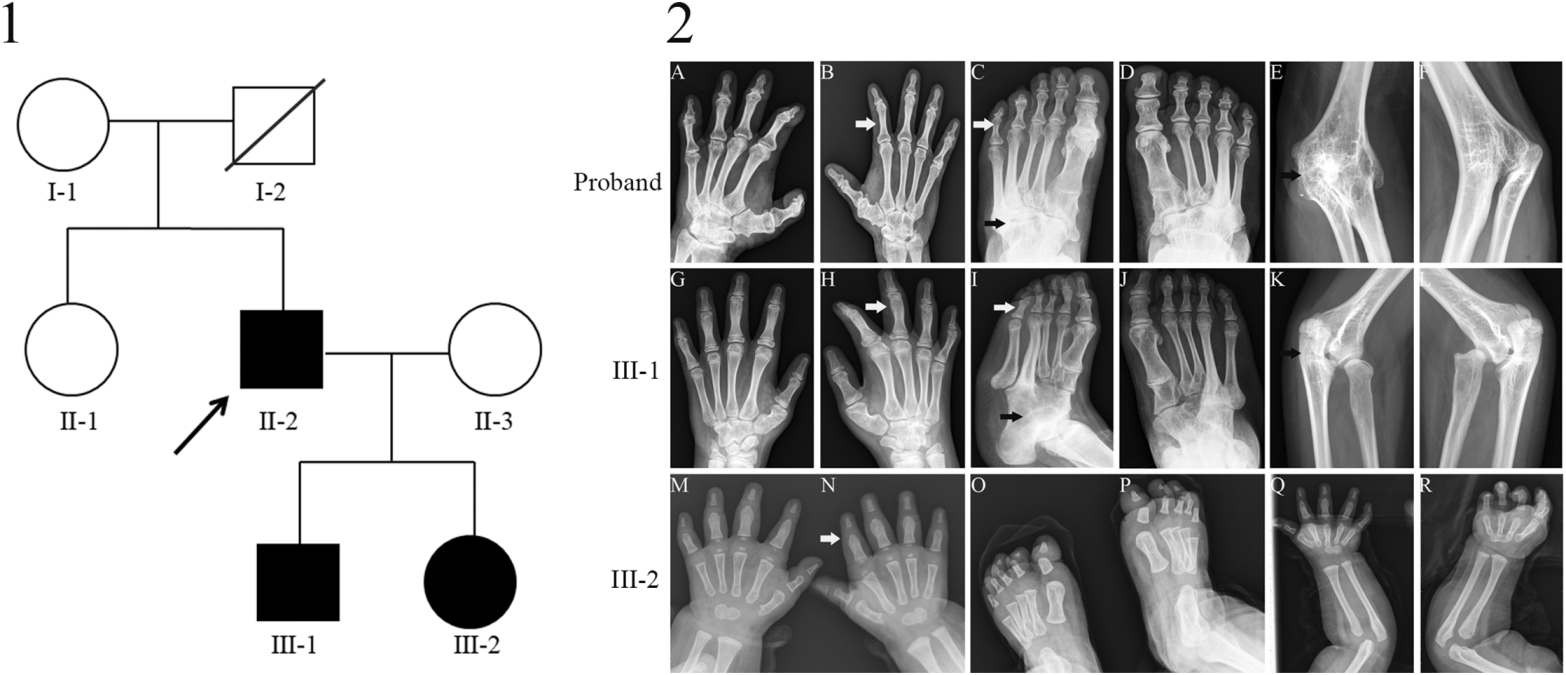
Family pedigree, and radiographs of the hands, feet, and elbows. (1) Pedigree of the family. Squares and circles represent males and females, respectively. The affected family members are indicated in black symbols, and the proband is indicated by an arrow. (2) Radiographs of the hands, feet, and elbows of individuals proband, III‐1, and III‐2, displaying the following abnormalities: lack of the middle and proximal interphalangeal joints in the 2nd–5th fingers and toes (white arrows); shortened proximal phalanx of the first toe and fused elbow joint, and tarsal fusion (black arrows). (A–F) proband. (G–L) III‐1. (M–R) III‐2.

The basic characteristics of the affected family members are displayed in Table 1, and the family pedigree is shown in Figure 1‐I. The father and son presented with features of BDA1, namely lack of the middle and proximal interphalangeal joints of the second, third, fourth, and fifth fingers and toes; shortened proximal phalanx of the first toe; fused tarsal bones; bone fusion in both elbow joints; and elbow valgus deformity. Additionally, all the fingers of the daughter lacked a bone germinal center (Figure 1‐II and Table 1).

**TABLE 1:** The basic characteristics of the affected family members.

Basic characteristics	Proband	III‐1	III‐2
Age, Y	47	11	1
Restricted elbow joints movement, Y	18	10	−
Gender	M	M	F
Proximal symphalangism	+	+	−
Distal symphalangism	−	−	−
Metacarpophalangeal synostosis	−	−	−
Synostosis of carpal bones	−	−	−
Synostosis of tarsal bones	+	+	−
Tarsometatarsal synostosis	−	−	−
Abnormalities of the second and third phalanx of the middle phalanx (brachydactyly type A1)	+	+	−
Hypoplastic/short the proximal phalanx of 1st toe	+	+	−
Absence of ossification center	−	−	+
Deafness	−	−	−
Pain (hand and/or feet)	−	−	NA
Sensory impairment (hand and/or feet)	−	−	NA
Numbness (hand and/or feet)	−	−	NA
Increased sweating (hand and/or feet)	−	−	NA

Abbreviations: F, female; M, male; NA, non‐applicable.

### 3.2 Variant detection

The exome‐capture statistics are shown in Table S1. In brief, the whole‐exome sequencing identified 130 411 variations, including 117 782 SNPs and 12 629 InDels. Among them, the numbers of frameshift deletions, frameshift insertions, non‐frameshift deletions, non‐frameshift insertions, stop gains, stop losses, and unknown variants were 57, 43, 157, 135, 4, 0, and 73, respectively.

### 3.3 Genetic and bioinformatic analyses

**FIGURE 2:**
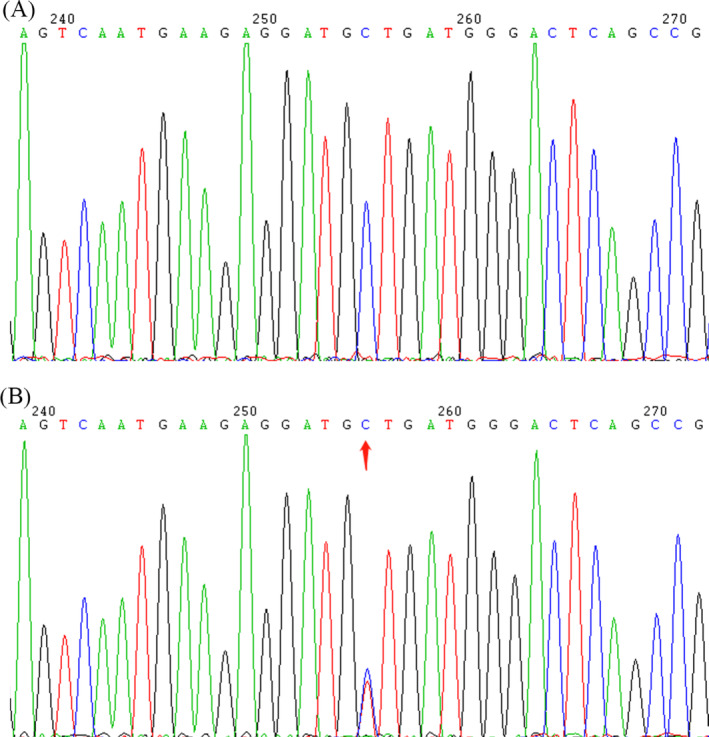
A variant in the GDF5 gene, arrow indicate the changed position. (A) GDF5 wild type (I‐1, II‐1, and II‐3); (B) GDF5 heterozygote (proband, III‐1, and III‐2).

A pre‐defined panel of brachydactyly‐related genes (*BMP2*, *BMPR1B*, *CHST11*, *CHSY1*, *GDF5*, *HDAC6*, *HOXD13*, *IHH*, *NOG*, *PDE3A*, *PITX1*, *PRMT7*, *PTHLH*, *ROR2*, *RUNX2*, *TBC1D24*, and *TRPV4*) were evaluated. *GDF5* was found to be a candidate pathogenic gene in the proband, who did not carry any other known brachydactyly‐related pathogenic variant. The variant was located on chromosome 20, position 34 021 789, G1424A, NM_000557, p.S475N, namely, rs121909347. This variant was analyzed for evolutionary conservation, and the results showed that it was located in a highly conserved region among multiple animal species (Table 2). Via multiple bioinformatics software, including SIFT, Polyphen2, LRT, FATHMM, and REVEL, the variant was predicted to perturb the protein function. At present, there are no relevant records about the variant frequency of this variant in major databases, such as 1000 g, esp6500, and GnomAD. Sanger sequencing the G1424A in *GDF5* of other family members revealed that the affected relatives (III‐1 and III‐2) have the same variant, and the non‐affected relatives (I‐1, II‐1, and II‐3) have no variant (Figure 2).

**TABLE 2:** Evolutionary conservation analysis for the variant in *GDF5.*

Protein Acc.	Gene	Organism	Amino acid sequences		
NP_00548.1	*GDF5*	*Homo.sapiens*	446	HAVIQTLMNSMDPESTPPTCCVPTRLSPISILFIDSANNVVYKQYEDMVV	495
XP_530287.2	*GDF5*	*Pan. troglodytes*	446	HAVIQTLMNSMDPESTPPTCCVPTRLSPISILFIDSANNVVYKQYEDMVV	495
XP_001099806.1	*GDF5*	*Macaca.mulatta*	444	HAVIQTLMNSMDPESTPPTCCVPTRLSPISILFIDSANNVVYKQYEDMVV	493
XP_542974.1	*GDF5*	*Canis.lupus*	444	HAVIQTLMNSMDPESTPPTCCVPTRLSPISILFIDSANNVVYKQYEDMVV	493
NP_001179202.1	*GDF5*	*Bos.taurus*	444	HAVIQTLMNSMDPESTPPTCCVPTRLSPISILFIDSANNVVYKQYEDMVV	493
NP_032135.2	*Gdf5*	*Mus.musculus*	440	HAVIQTLMNSMDPESTPPTCCVPTRLSPISILFIDSANNVVYKQYEDMVV	489
XP_003749648.1	*Gdf5*	*Rattus.norvegicus*	440	HAVIQTLMNSMDPESTPPTCCVPTRLSPISILFIDSANNVVYKQYEDMVV	489
XP_989669.1	*GDF5*	*Gallus. gallus*	445	HAVIQTLMNSMDPESTPPTCCVPTRLSPISILFIDSANNVVYKQYEDMVV	494
XP_002662587.1	*gdf5*	*Danio.rerio*	419	HAIIQTLMNSMDPRSTPPTCCVPTRLSPISILYIDSANNVVYKQYEDMVV	468
NP_001128589.1	*gdf5*	*Xenopus.tropicalis*	441	HAVIQTLMNSMDPETTPPTCCVPTRLSPISILYTDSANNVVYKQYEDMVV	490

*Note*: Proband GDF5:NM_000557:exon2:c.G1424A:p.S475N.

### 3.4 Prediction of the protein structure

**FIGURE 3:**
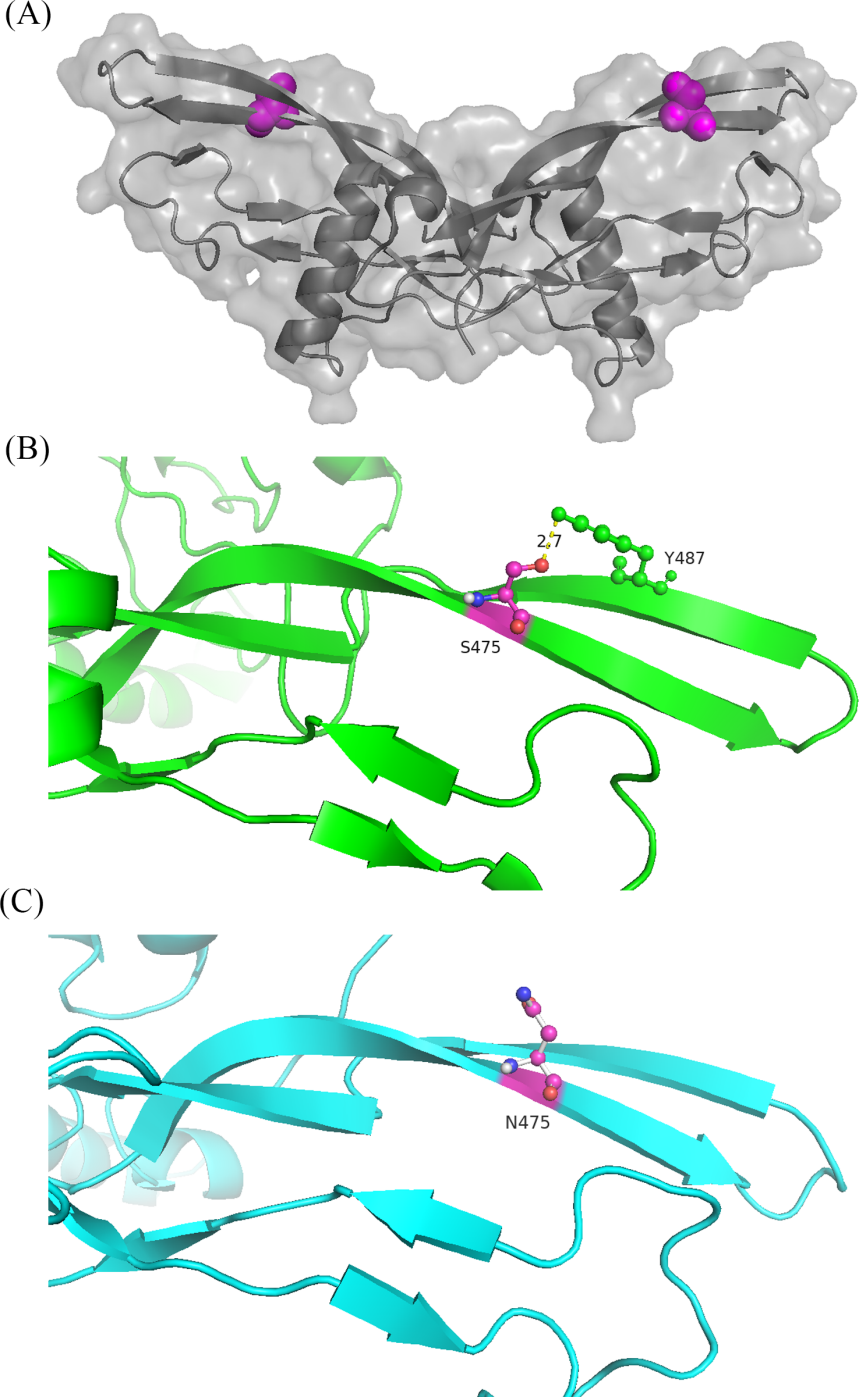
3D presentation and structural comparison of the wild‐type and mutant (S475N) GDF5 proteins. (A) 3D presentation of the GDF5 protein (prepared and visualized using I‐TASSER and PyMol, respectively). The S475N variant is highlighted in pink. (B) The GDF5 S475 polypeptide. (C) The GDF5 N475 polypeptide.

The tertiary structures of the wild‐type and mutant GDF5 proteins were predicted using I‐TASSER (Figure 3). The S475N variant is highlighted on the 3D model of GDF5 (Figure 3A). GDF5 S475N is located within the finger of the GDF5 dimer. The substitution of the negatively charged amino acid S475 with the neutral N475 disrupts the formation of salt bridges with Y487 (Figure 3B, C). Therefore, the GDF5 S475N variant destroys the connection between S475 and Y487, presumably impairing the stability, structure, and function of the protein and causing its degradation.

## 4 DISCUSSION

Here, we described a novel *GDF5* variant in a Chinese family with BDA1 and SYNS2. Our whole‐exome sequencing revealed that the proband had a variant in the *GDF5* gene. The variant was located within the functional region, and the mutated residue was found to be highly conserved among species. Via bioinformatic analyses, we predicted this variant to be deleterious, which perturb the protein function. This variant disrupts the binding site of GDF5 at the 487th residue. The stability of the protein and the affinity of the protein for its protein may be changed, consequently, the abnormalities in cartilage and bone development ensue.

The proband analyzed here presented with carpal/tarsal fusion, proximal interphalangeal joint adhesion, humerus radius connection, and progressive conductive deafness, which are characteristic features of SYNS. To date, the molecular mechanism underlying this progressive chondrogenic and osteoblastic disorder has remained unknown.

The S475N variant on the GDF5 gene has already been described in an Iranian family with tarsal‐carpal fusion, humeroradial synostosis, brachydactyly, and proximal symphalangism, which has been also mentioned and included in the OMIM database (MIM 610017).^19^ Elisa Degenkolbe et al. had found that BDA1 and SYNS2 are caused by GDF5 W414R through two separate molecular mechanisms, one involving BMPR1A and the other involving NOG.^16^ Variants in *GDF5* or *NOG* lead to similar phenotypes in SYM1 and SYNS1/2. The same authors have found that when NOG is present, GDF5 W414R lost BMPR1A signal and increased the BMPR1B signal. GDF5 W414R has reduced sensitivity to NOG. It is worth mentioning that the decreased interaction between GDF5 W414R and BMPR1A is not actually neutralized by the decreased sensitivity to NOG but resulted in the imbalance of BMPR1A and BMPR1B signals. Schwaerzer et al. found that GDF5‐S94N exhibits impaired binding to BMPRII causing alleviated Smad and non‐Smad signaling and reduced chondrogenic differentiation of ATDC5 cells; however, chondrogenesis in mouse micromass cultures was strongly enhanced by GDF5‐S94N.^17^ These data show how variants in the *GDF5* gene affect the bone development in the hands and feet and classify the disease as a specific type of brachyal deformity and SYNS2.

The topology of the GDF5 monomer comprises two β‐sheets forming the fingers, this serine residue (GDF5‐S94N) is located in the β‐sheet β6 of the knuckle epitope, which consists of β‐sheets β2‐β4 of finger 1 and β6 and β7 of finger 2 forming a convex surface, and it interacts with the concave surface of BMP type II receptors.^17^ However, the serine residue (GDF5‐S475N) is located in β‐sheet of finger 2 of the GDF5 monomer, moreover, the variant (GDF5‐S475N) interferes with the BMP type I receptor (BMPR1A and BMPR1B) binding interface^16^; therefore, the variant (GDF5‐S475N) may have the characteristics both GDF5‐S94N and GDF5‐S414R, and we speculated that serine 475 of GDF5 to be important for the propagation of the GDF5‐induced signaling and for the interaction of GDF5 with NOG and the C‐terminal part of the clip region of BMPRI and BMPRII.

This association of the *GDF5* S475N variant with BDA1 and SYNS2 revealed a dual pathomechanism concurrently involving gain and loss of function. It was predicted that the reduced affinity of GDF5‐S475N to the BMP/GDF antagonist noggin averts inhibition by noggin, and the resulting gain of function causes the SYNS2 phenotype, while reduced signaling downstream of the BMP receptor causes the BDA1 phenotype. However, further research is needed to improve the understanding of the pathogenesis.

In summary, we speculated the unique pathomechanism caused by GDF5‐S475N, and this is the first report of this variant, identified in a Chinese family with BDA1 and SYNS2. Additionally, it could be applied for genetic counseling.

## AUTHOR CONTRIBUTIONS

Juyi Li and Jianxin Liu conceived and designed the experiments. Juyi Li, Jianxin Liu, Aiping Deng, Lei Fu, and Pei Yang performed the experiments. Pei Yang, Xiaofei Jian, Juyi Li, Chao Liu, and Jianxin Liu analyzed the data. Xiaofang Liang and Juyi Li wrote the paper. Juyi Li, Xiaofang Liang, and Xiufang Wang contributed equally. All authors contributed to the article and approved the submitted version.

## FUNDING INFORMATION

This study was supported by Grants from the Central Guiding Local Science and Technology Development Special Project (No. 2022BGE272), and Guangdong Yiyang Healthcare Charity Foundation (JZ2022011).

## CONFLICT OF INTEREST STATEMENT

The authors declare that there is no duality of interest associated with this manuscript.

## Supporting information


**Table S1.** Details of WES.

## References

[1] Chang SC , Hoang B , Thomas JT , et al. Cartilage‐derived morphogenetic proteins. New members of the transforming growth factor‐beta superfamily predominantly expressed in long bones during human embryonic development. J Biol Chem. 1994;269:28227‐28234. 7961761

[2] Buxton P , Edwards C , Archer CW , Francis‐West P . Growth/differentiation factor‐5 (GDF‐5) and skeletal development. J Bone Joint Surg Am. 2001;83‐A(Suppl 1):S23‐S30. 11263662

[3] Kotzsch A , Nickel J , Seher A , Sebald W , Muller TD . Crystal structure analysis reveals a spring‐loaded latch as molecular mechanism for GDF‐5‐type I receptor specificity. EMBO J. 2009;28:937‐947. 19229295 10.1038/emboj.2009.37 PMC2670865

[4] Miyazono K , Kamiya Y , Morikawa M . Bone morphogenetic protein receptors and signal transduction. J Biochem. 2010;147:35‐51. 19762341 10.1093/jb/mvp148

[5] Lories RJ , Luyten FP . Bone morphogenetic protein signaling in joint homeostasis and disease. Cytokine Growth Factor Rev. 2005;16:287‐298. 15993360 10.1016/j.cytogfr.2005.02.009

[6] Seemann P , Schwappacher R , Kjaer KW , et al. Activating and deactivating mutations in the receptor interaction site of GDF5 cause symphalangism or brachydactyly type A2. J Clin Invest. 2005;115:2373‐2381. 16127465 10.1172/JCI25118 PMC1190374

[7] Everman DB , Bartels CF , Yang Y , et al. The mutational spectrum of brachydactyly type C. Am J Med Genet. 2002;112:291‐296. 12357473 10.1002/ajmg.10777

[8] Degenkolbe E , Schwarz C , Ott CE , et al. Improved bone defect healing by a superagonistic GDF5 variant derived from a patient with multiple synostoses syndrome. Bone. 2015;73:111‐119. 25543012 10.1016/j.bone.2014.12.017

[9] Li Q , Bai F , Chen S . Frameshift mutation in a Chinese patient with brachydactyly type C involving the third metacarpal: a case report. Orthopaedic Surgery. 2022;14:2386‐2390. 35819086 10.1111/os.13383 PMC9483038

[10] Faryal S , Farooq M , Abdullah U , et al. A GDF5 frameshift mutation segregating with grebe type chondrodysplasia and brachydactyly type C+ in a 6 generations family: clinical report and mini review. Eur J Med Genet. 2021;64:104226. 33872773 10.1016/j.ejmg.2021.104226

[11] Genovesi ML , Guadagnolo D , Marchionni E , et al. GDF5 mutation case report and a systematic review of molecular and clinical spectrum: expanding current knowledge on genotype‐phenotype correlations. Bone. 2021;144:115803. 33333243 10.1016/j.bone.2020.115803

[12] Seemann P , Brehm A , Konig J , et al. Mutations in GDF5 reveal a key residue mediating BMP inhibition by NOGGIN. PLoS Genet. 2009;5:e1000747. 19956691 10.1371/journal.pgen.1000747 PMC2776984

[13] Mundlos S . The brachydactylies: a molecular disease family. Clin Genet. 2009;76:123‐136. 19790289 10.1111/j.1399-0004.2009.01238.x

[14] Byrnes AM , Racacho L , Nikkel SM , et al. Mutations in GDF5 presenting as semidominant brachydactyly A1. Hum Mutat. 2010;31:1155‐1162. 20683927 10.1002/humu.21338

[15] Stange K , Thieme T , Hertel K , et al. Molecular analysis of two novel missense mutations in the GDF5 proregion that reduce protein activity and are associated with brachydactyly type C. J Mol Biol. 2014;426:3221‐3231. 25092592 10.1016/j.jmb.2014.07.029

[16] Degenkolbe E , Konig J , Zimmer J , et al. A GDF5 point mutation strikes twice—causing BDA1 and SYNS2. PLoS Genet. 2013;9:e1003846. 24098149 10.1371/journal.pgen.1003846 PMC3789827

[17] Schwaerzer GK , Hiepen C , Schrewe H , et al. New insights into the molecular mechanism of multiple synostoses syndrome (SYNS): mutation within the GDF5 knuckle epitope causes noggin‐resistance. J Bone Miner Res. 2012;27:429‐442. 21976273 10.1002/jbmr.532

[18] Shao Y , Zhao T , Zhang W , et al. Presence of the apolipoprotein E‐epsilon4 allele is associated with an increased risk of sepsis progression. Sci Rep. 2020;10:15735. 32978453 10.1038/s41598-020-72616-0 PMC7519096

[19] Dawson K , Seeman P , Sebald E , et al. GDF5 is a second locus for multiple‐synostosis syndrome. Am J Hum Genet. 2006;78:708‐712. 16532400 10.1086/503204 PMC1424701

