# Supplementary material for: A missense GDF5 variant causes brachydactyly type A1 and multiple‐synostoses syndrome 2
Source: JOR Spine. 2023 Nov 28;7(1):e1302. doi: 10.1002/jsp2.1302 (PMC10782059; doi:10.1002/jsp2.1302)
Supplement: Supplementary file 1 — Table S1. Details of WES. [file JSP2-7-e1302-s001.docx]

**Supplemental Table**. Details of WES

| **Exome Capture Statistics** | **Proband** |
| --- | --- |
| Total | 68,021,258 (100%) |
| Duplicate | 17,100,872 (25.14%) |
| Mapped | 67,976,928 (99.93%) |
| Properly mapped | 67,287,390 (98.92%) |
| PE mapped | 67,944,776 (99.89%) |
| SE mapped | 64,304 (0.09%) |
| With mate mapped to a different chr | 382,720 (0.56%) |
| With mate mapped to a different chr ((mapQ>=5)) | 333,239 (0.49%) |
| Initial_bases_on_target | 60,456,963 |
| Initial_bases_near_target | 75,840,481 |
| Initial_bases_on_or_near_target | 136,297,444 |
| Total_effective_yield (Mb) | 10,128.37 |
| Effective_sequences_on_target (Mb) | 6,144.30 |
| Effective_sequences_near_target (Mb) | 2,300.33 |
| Effective_sequences_on_or_near_target (Mb) | 8,444.64 |
| Fraction_of_effective_bases_on_target | 60.66 % |
| Fraction_of_effective_bases_on_or_near_target | 83.38 % |
| Average_sequencing_depth_on_target | 102 |
| Average_sequencing_depth_near_target | 30.33 |
| Mismatch_rate_in_target_region | 0.36 % |
| Mismatch_rate_in_all_effective_sequence | 0.47 % |
| Base_covered_on_target | 60,276,359 |
| Coverage_of_target_region | 19.70 % |
| Base_covered_near_target | 73,888,745 |
| Coverage_of_flanking_region | 97.43 % |
| Fraction_of_target_covered_with_at_least_10x | 97.16 % |
| Fraction_of_target_covered_with_at_least_50x | 75.86 % |
| Fraction_of_target_covered_with_at_least_100x | 44.23 % |
| Fraction_of_flanking_region_covered_with_at_least_10x | 70.54 % |
| Fraction_of_flanking_region_covered_with_at_least_50x | 19.77 % |
| Fraction_of_flanking_region_covered_with_at_least_100x | 4.47 % |
